# Effects of rhubarb peony decoction combined with antibiotics in treating pediatric periappendiceal abscess

**DOI:** 10.3389/fped.2023.1112034

**Published:** 2023-03-29

**Authors:** Zhixiong Lin, Huiping Zeng, Shujie Cai, Fei Chen, Xiang Wang, Dianming Wu, Mingkun Liu, Yifan Fang

**Affiliations:** ^1^ Department of Pediatric Surgery, Fujian Children's Hospital (Fujian Branch of Shanghai Children's Medical Center), College of Clinical Medicine for Obstetrics & Gynecology and Pediatrics, Fujian Medical University, Fuzhou, China; ^2^ Department of Pediatric Surgery, Fujian Maternity and Child Health Hospital, College of Clinical Medicine for Obstetrics & Gynecology and Pediatrics, Fujian Medical University, Fuzhou, China

**Keywords:** periappendiceal abscess, rhubarb peony decoction, traditional Chinese medicine, conservative treatment, appendectomy

## Abstract

**Background/purpose:**

Rhubarb peony decoction (RPD) is a formula of traditional Chinese medicine that has been widely used to treat intra-abdominal inflammatory diseases. To investigate the therapeutic efficacy of RPD in pediatric periappendiceal abscess, patients who received intravenous antibiotics alone were compared with those treated with intravenous antibiotics combined with RPD.

**Methods:**

A retrospective review of children with periappendiceal abscess who received conservative treatment in our hospital between January 2013 and April 2022 was performed. The patients were divided into an intravenous antibiotic group (the control group) and an intravenous antibiotic combined with RPD group (the intervention group). Interval appendectomy (IA) was generally performed 10–12 weeks after conservative treatment. The primary outcome was the cure rate of conservative treatment, while the secondary outcomes included the recurrence rate, days of total intravenous antibiotic use, length of hospital stay (LOS), postoperative complications, and liver injury caused by RPD.

**Results:**

A total of 142 patients (77 girls and 65 boys) were included, 52 in the control group and 90 in the intervention group. The two groups were similar in demographic data and clinical characteristics (*P* > 0.05). The mean total course of RPD in the intervention group was 11.82 days. The intervention group had a significantly higher cure rate than the control group (93.33% vs. 80.77%, *P* = 0.029), and the length of total intravenous antibiotic use (*P* = 0.150), LOS (*P* = 0.077), recurrence rate (9.52% vs. 4.76%, *P* = 0.439), as well as the operation time (*P* = 0.101), LOS (*P* = 0.572), and postoperative complications (*P* = 0.549) were not significantly different between the two groups when the patients received IA. No patient had a liver injury caused by RPD during the treatment.

**Conclusion:**

Intravenous antibiotics combined with RPD demonstrated high effectiveness and safety for treating pediatric periappendiceal abscess.

## 1. Introduction

The incidence of periappendiceal abscess accounts for 9.7% of complex appendicitis in the pediatric population (1). However, its optimal treatment (surgery or conservative treatment) is still controversial (2). Although recent guidelines have recommended units with skilled laparoscopy to perform an immediate emergency appendectomy to shorten the hospital stay (3), considering the high incidence of complications in emergency surgery, conservative management (antibiotics with or without percutaneous drainage) is still the preference of most doctors (4, 5).

Traditional Chinese medicine (TCM) has been widely used in Asian countries with a long history. Recent studies have shown that TCM plays an important role in treating coronavirus disease 2019 (COVID-19) and controlling mild hypertension (6, 7). Meanwhile, rhubarb peony decoction (RPD) is one of the most famous TCM formulas in China. From the Eastern Han Dynasty, RPD (also called “Dahuang-Mudan decoction”) has been extensively used to treat intra-abdominal inflammatory diseases, such as appendicitis, pancreatitis, and inflammatory bowel disease, usually achieving satisfactory effectiveness (8, 9). However, most of these studies focus on adults, and the use of RPD in treating periappendiceal abscess in children is rarely reported. To this end, the present study aimed to evaluate the effect of RPD by comparing the therapeutic effects of intravenous antibiotics alone and antibiotics combined with RPD on pediatric periappendiceal abscess.

## 2. Materials and methods

### 2.1. Patient samples

A retrospective review was carried out from January 2013 to April 2022 in pediatric patients (aged between 3 and 14 years) who were diagnosed with periappendiceal abscess and received conservative treatment. Children having undergone an emergency appendectomy or who accepted percutaneous drainage within 24 h after admission and those complicated with intestinal obstruction, hepatic or renal dysfunction, coagulation disorders, and allergy to antibiotics were excluded. The children were divided into a control group (C-group) and an intervention group (I-group) according to different treatment approaches. The C-group was treated with intravenous antibiotics alone, while the I-group was treated with intravenous antibiotics combined with oral RPD. This study strictly adhered to the tenets of the Declaration of Helsinki and was approved by the ethics committee at Fujian Children's Hospital (No. 2022ETKLR08023).

### 2.2. Intravenous antibiotics and oral RPD treatments

The patients were diagnosed with periappendiceal abscess by physical examination, laboratory examination, ultrasound (US), or abdominal computed tomography (CT). Those in the C-group received intravenous antibiotic therapy (50 mg/kg ceftriaxone and 30 mg/kg metronidazole daily) immediately after admission, and the course of antibiotic treatment lasted for 7–14 days, while the I-group patients were treated with intravenous antibiotics combined with oral RPD. The choice of receiving TCM treatment mainly depended on the opinions of surgeons and the consent of the parents of patients. Children began taking oral RPD within 24 h after admission, and the treatment lasted at least 1 week. RPD is composed of Rhei Radix Et Rhizoma [the dried root and rhizome of *Rheum alexandrae* Batalin (Polygonaceae)] da Huang (Huangtai Chinese medicine decoction Piece Technology Co. Ltd., Anhui, China), Moutan cortex [the dried root cortex of *Paeonia* × *suffruticosa* Andrews (Paeoniaceae)], mu dan pi (Yanlaifu Pharmaceutical Co,. Ltd., Xiamen, China), semen persicae [the dried semen of *Prunus persica* (L.) Batsch (Rosaceae)], tao ren (Yonggang decoction Piece Factory Co. Ltd., Haozhou, China), waxgourd seed [the seeds from *Benincasa hispida* (Thunb.) Cogn. (Cucurbitaceae)], dong gua ren (Yonggang decoction Piece Factory Co. Ltd., Haozhou, China), and Natrii sulfas (mang xiao, a mineral, Na_2_SO_4_·10H_2_O, Huarun Sanjiu Medicine Co. Ltd., Guangdong, China). The prescription was provided by TCM physicians based on the syndrome differentiation of the children and decocted by the hospital pharmacy. The decoction was taken orally 0.5–1.0 h after meals, twice daily in two doses of 50 ml, and the treatment period was 7–14 days.

Routine blood examination, C-reactive protein (CRP) investigation, and appendix area US were performed at an interval of 3 days to evaluate the treatment effect. Blood biochemistry tests were performed after oral administration of RPD for a week to evaluate whether impairment of liver function developed. The size of the periappendiceal abscess was estimated by US for the largest anteroposterior and lateral size to give the greatest two-dimensional area on an axial image. If the abscess remained unchanged or gradually increased during treatment, or if the abdominal pain and laboratory examination had worsened, the conservative treatment was considered to have failed, and percutaneous puncture drainage or surgical treatment would then be adopted. Patients requiring appendectomy or those with recurrence within 7 days after discharge were also considered to have encountered a conservative treatment failure. Patients were discharged from the hospital if the following criteria were met: children experienced no abdominal pain and had a normal diet, the laboratory examination was significantly improved, and the maximum diameter of the abscess was less than 3 cm. Then, oral antibiotics could be changed after discharge. Interval appendectomy (IA) was generally performed 10–12 weeks after discharge, and all patients were followed up for at least 3 months after IA.

The primary outcome of this study was the cure rate of conservative treatment, while the secondary outcomes included the recurrence rate, length of total intravenous antibiotic use, length of hospital stay (LOS), postoperative complications, and liver injury caused by RPD.

### 2.3. Statistical analysis

The data were analyzed using SPSS 21.0 statistical software. Characteristics were compared between the two groups using Student's *t*-test for continuous variables and Fisher's exact test for categorical variables. A *P*-value < 0.05 was considered a statistically significant difference.

## 3. Results

**Figure 1:**
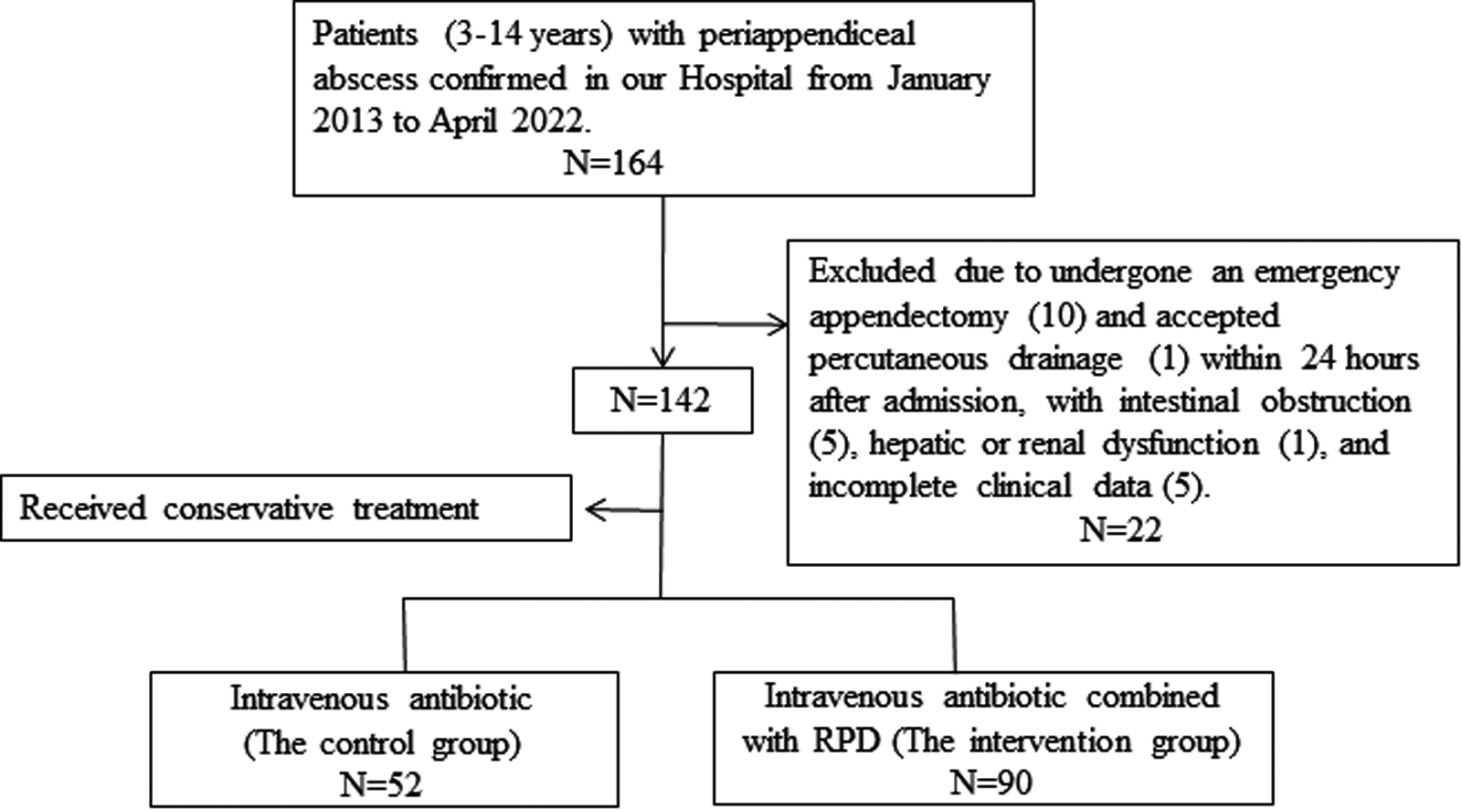
Study flowchart.

A total of 142 patients (77 girls and 65 boys) diagnosed with periappendiceal abscess and having received conservative treatment were enrolled, 52 in the C-group and 90 in the I-group (a flowchart of the study is shown in Figure 1). The groups were similar in their gender (*P =* 0.082), age (*P* = 0.301), weight (*P* = 0.505), and duration of symptoms (*P* = 0.310). The abscess size at admission (25.85 ± 16.68 cm^2^ vs. 26.71 ± 13.99 cm^2^; *P* = 0.744), WBC count (16.93 ± 6.06 × 10^9^/L vs.18.72 ± 6.11 × 10^9^/L; *P* = 0.095), and CRP level (62.14 ± 29.35 vs. 73.67 ± 50.28; *P* = 0.133) were also similar between the two groups. The demographics and clinical characteristics of the patients in the two groups before treatment are presented in Table 1.

**Table 1:** Patient demographics and clinical characteristics of the control group and intervention group before treatment.

Characteristics	C-group (*n* = 52)	I-group (*n* = 90)	*P* ^a^
Sex (F/M)	23/29	54/36	0.082
Median age (years)	5 (3–12)	5 (3–13)	0.301
Weight (kg)	19.05 (11.5–42.00)	17.25 (11.00–52.00)	0.505
Duration of symptoms (day)	5 (1–15)	6.5 (1–14)	0.310
Abscess size at admission (cm^2^)	25.85 ± 16.68	26.71 ± 13.99	0.744
WBC count (10^9^/L)	16.93 ± 6.06	18.72 ± 6.11	0.095
CRP level (mg/L)	62.14 ± 29.35	73.67 ± 50.28	0.133

C-group, control group; I-group, intervention group; WBC, white blood cell; CRP, C-reactive protein.

Data were expressed as mean ± SD or median (range: minimum–maximum).

^a^ Fisher’s exact test for nominal categories and *t*-test for continuous variables.

There were no significant differences in the length of total intravenous antibiotic use (12.96 ± 4.69 days vs. 14.02 ± 3.88 days, *P* = 0.150) and LOS (10.69 ± 4.11 days vs. 11.85 ± 3.52 days, *P* = 0.077) between the two groups. The mean total course of RPD in the I-group was 11.82 days. Compared with the C-group, the I-group had a significantly higher cure rate (93.33% vs. 80.77%, *P* = 0.029), and the time to treatment failure was earlier in the C-group (median 4.0 days vs. 5.5 days, *P* = 0.008). Laparoscopic appendectomy was performed on all 16 patients who received unsuccessful treatment. During the entire treatment and postoperative follow-up, no patients had a liver injury caused by RPD (Table 2).

**Table 2:** Outcomes of conservative treatment for the control group and intervention group after treatment.

Characteristics	C-group (*n* = 52)	I-group (*n* = 90)	*P*
Total course of RPD (days)	0	11.82 ± 3.03	<0.001
Total intravenous antibiotics (days)	12.96 ± 4.69	14.02 ± 3.88	0.150
Cure rate, *n* (%)	42 (80.77%)	84 (93.33%)	0.029
Failure rate of treatment, *n* (%)	10 (19.23%)	6 (6.67%)	
Time to treatment failure (days)	4.0 (2–5)	5.5 (4–10)	0.008
Treatment after failure			
LOS (days)	10.69 ± 4.11	11.85 ± 3.52	0.077
RPD-induced liver injury	0	0	1.000
Recurrence rate*, *n* (%)	4 (9.52%)	4 (4.76%)	0.439

RPD, rhubarb peony decoction; LOS, length of hospital stay.

^a^ Patients with treatment failure were excluded; cure rate = (number of people cured/total number) × 100%.

Among those who succeeded in conservative treatment, four patients developed recurrent appendicitis in both groups, and no significant difference (9.52% vs. 4.76%, *P* = 0.439) was observed in the recurrence rate between the two groups. The median time appearing relapse was 35 (9–53) days in the C-group and 30 (15–45) days in the I-group (*P* > 0.05). Among the recurrent patients, two chose to continue nonoperative management, while six received emergency laparoscopic appendectomy (*P =* 1.000). In addition, no significant difference was observed in the operation time (98.75 ± 37.24 min vs. 89.50 ± 37.92 min, *P* = 0.740) and the length of hospital stay (9.25 ± 4.57 days vs. 11.25 ± 5.85 days, *P* = 0.610). Additionally, most of the recurrent patients were found to be girls (F:M = 7:1) (Table 3).

**Table 3:** Outcomes of patients who showed recurrence for the control group and intervention group after treatment.

Characteristics	C-group (*n* = 4)	I-group (*n* = 4)	*P*
Sex (F/M)	4/0	3/1	1.000
Interval time of recurrence (days)	35 (9–53)	30 (15–45)	0.822
Treatment after recurrence			1.000
Conservative treatment	1	1	
Surgery	3	3	
Operative time (min)	98.75 ± 37.24	89.50 ± 37.92	0.740
LOS (days)	9.25 ± 4.57	11.25 ± 5.85	0.610

LOS, length of hospital stay.

Finally, 107 patients received interval appendectomy and completed the follow-up, except for 11 (3 in C-group and 8 in I-group) who did not return to the hospital for surgery within 12 weeks. The operation time (61.40 ± 15.18 min vs. 67.90 ± 20.68 min, *P* = 0.101), length of stay (5.51 ± 1.99 days vs. 5.73 ± 1.85 days, *P* = 0.572), and rate of postoperative complications (2.86% vs. 1.39%, *P* = 0.549) were not significantly different between the two groups. One patient had an intestinal obstruction in the C-group, and one developed pelvic fluid collection. Both of them were discharged after conservative treatment (Table 4).

**Table 4:** Outcomes of interval appendectomy after conservative treatment for the control group and intervention group.

Characteristics	C-group (*n* = 35)^a^	I-group (*n* = 72)^a^	*P*
Operative time (min)	61.40 (15.18)	67.90 (20.68)	0.101
LOS (days)	5.51 (1.99)	5.73 (1.85)	0.572
Postoperative complications, *n*(%)	1 (2.86%)	1 (1.39%)	0.549

LOS, length of hospital stay.

^a^ Patients who showed treatment failure and recurrence were excluded.

## 4. Discussion

Appendicitis is the most common acute abdominal disease in the pediatric population. Due to the delay in diagnosis and stercolith in the appendix cavity, it often contributes to perforation and periappendiceal abscess formation, accounting for 3% of appendicitis cases (10). In this situation, emergency surgery may be difficult since the appendix and surrounding tissues are edematous and fragile. The risk of intestinal resection is up to 25%, and that of complications is 57% (11). However, the success rate of conservative treatment can reach 90% (12), and there are fewer complications, even without an IA, after conservative management. In this case, conservative treatment is accepted by most doctors and parents, which includes antibiotics with or without percutaneous drainage (4, 13). Double-agent antibiotic therapy can provide equal efficacy and is more cost-effective than triple-agent therapy in the case of treating periappendiceal abscess. Additionally, percutaneous drainage is required when the treatment is ineffective (14, 15). For children, CT guidance percutaneous drainage needs anesthesia and increases the risk of radiation exposure, and some studies recognize drainage as a risk factor for the recurrence of appendicitis (16, 17). To this end, considering the treatment characteristics of TCM, antibiotics combined with RPD are often adopted by our institute for the conservative treatment of periappendiceal abscess, and favorable effects are achieved.

In China, TCM plays a major role in the conservative treatment of inflammatory diseases. Chinese people and their children are more willing to accept TCM treatment and have a high recognition of its efficacy. As early as 1979, Nan Kai Hospital in China reported using Chinese and Western medicine to treat acute abdominal diseases, including acute appendicitis, intestinal obstruction, pancreatitis, and biliary tract infection. Among 1,200 patients with acute appendicitis, 130 cases of periappendiceal abscess were treated with integrated traditional Chinese and Western medicine. The total cure rate was 94.2%, and the recurrence rate was 14.5% (18). However, the report did not provide details of the TCM prescriptions for treating periappendiceal abscess and the distribution of the included patients (adults and children). The cure rate of antibiotics combined with RPD in the present study was 93.33%, which was similar to that reported by Nan Kai Hospital and was significantly higher than that of intravenous antibiotics alone. However, the recurrence rate after successful conservative treatment was 4.76% in the I-group, lower than the reported 14.5%, which might be attributed to the different treatment strategies, such as the types of TCM and antibiotics. During the follow-up, the operation time, LOS, and the incidence of postoperative complications were similar between the two groups after receiving interval appendectomy. In recent years, an increasing number of traditional medicines (including traditional Japanese medicine) have achieved satisfactory results in the treatment of pediatric surgical diseases, such as perianal abscess (19), biliary atresia (20), and spermatic cord hydrocele (21).

In TCM, the patients can be categorized according to the principles of syndrome differentiation. Periappendiceal abscess is also called “intestinal carbuncle” and belongs to the accumulated damp-heat syndrome. The treatment should be based on the principle of heat-clearing and blood circulation promotion to remove the meridian obstruction. Rhubarb and moutan bark are the main components of RPD, which are also known as the monarch herb (Jun herb) in the TCM theory. Rhubarb provides the functions of heat-clearing, fire-expelling, blood-cooling, and toxin-relieving. Moutan bark is endowed with the functions of promoting blood circulation to dissipate blood stasis, heat-clearing, and detoxifying. The therapeutic effect will be more effective in the case of a drug combination. The minister drug (Chen herb), such as prunus persica, wax gourd kernel, and mirabilite, helps strengthen the curative effect of the monarch drug or is generally used to treat accompanying symptoms.

According to the opinion of contemporary medical research, the above therapeutic effects are attributed to the main bioactive constituents of RPD. The main bioactive constituents extracted from the RPD include emodin, aloe-emodin, rhein, paeoniflorin, and amygdalin, which have been shown to have extensive anti-inflammatory and antioxidant activities in modern pharmacological studies (22–25). Nong et al. confirmed that RPD dealt with colitis by restoring the balance of multiple disturbed pathways and regulating to a normal condition (26). Apart from efficacy, the safety of TCM is another factor to be considered in the treatment. There are reports that TCM may induce liver injury (27). In this study, the average number of days of oral RPD was 10.48, and no RPD-induced liver damage was found during the treatment, suggesting the safety of taking RPD for the treatment of periappendiceal abscess in the short term.

There are some limitations in our study. First, this is a single-center retrospective study with limited sample size. In addition, it may be difficult for children to receive traditional medicine formulations because of the uncomfortable taste. Finally, although antibiotics combined with RPD are found to be capable of improving the success rate in the treatment of pediatric periappendiceal abscess, the RPD includes several different herbs, such as Rhei Radix Et Rhizoma, Moutan cortex, semen persicae, waxgourd seed, and Natrii sulfas, and which chemical substance plays a key role in this pathology process and its complex composition make the specific mechanism still unclear.

## 5. Conclusion

To the best of our knowledge, this study was the first report on the effect of RPD in children with periappendiceal abscess and showed that intravenous antibiotics combined with RPD demonstrated high effectiveness and safety for the treatment of pediatric periappendiceal abscess. Given the limitations of this study, the efficacy of RPD in treating pediatric periappendiceal abscess needs to be further justified by a prospective randomized study in the future.

## Data Availability

The raw data supporting the conclusions of this article will be made available by the authors, without undue reservation.
